# Endocan as a Potential Marker for Predicting All-Cause Mortality in Hemodialysis Patients

**DOI:** 10.3390/jcm12237427

**Published:** 2023-11-30

**Authors:** Jia-Hong Lin, Bang-Gee Hsu, Chih-Hsien Wang, Jen-Pi Tsai

**Affiliations:** 1 Department of Internal Medicine, Dalin Tzu Chi Hospital, Buddhist Tzu Chi Medical Foundation, Chiayi 62247, Taiwan; leo77119@gmail.com; 2 Division of Nephrology, Hualien Tzu Chi Hospital, Buddhist Tzu Chi Medical Foundation, Hualien 97004, Taiwan; gee.lily@msa.hinet.net (B.-G.H.); wangch33@gmail.com (C.-H.W.); 3 School of Medicine, Tzu Chi University, Hualien 97004, Taiwan; 4 Division of Nephrology, Department of Internal Medicine, Dalin Tzu Chi Hospital, Buddhist Tzu Chi Medical Foundation, Chiayi 62247, Taiwan

**Keywords:** endocan, creatinine, end-stage renal disease, hemodialysis, mortality

## Abstract

Endocan, a pro-inflammatory cytokine and pro-angiogenic factor, is a marker of endothelial dysfunction and has been proven to correlate with cardiovascular disease. In hemodialysis (HD) patients, cardiovascular disease is the major cause of mortality. Our study aimed to investigate the relationship between serum endocan and all causes of mortality in HD patients. A total of 103 patients, aged over 20 years old and undergoing HD for more than 3 months, were included and followed for 36 months. Mortality events, serum endocan, biochemical data, body mass index, systolic and diastolic blood pressure, baseline characteristics, and the use of antihypertensive and lipid-lowering drugs were recorded. In our study, a total of 26 deaths (25.2%) occurred. Hemodialysis patients with diabetes mellitus, older age, higher serum endocan, and lower creatinine and albumin levels had a higher risk of mortality. Adjusting for prognostic variables, HD patients with higher serum endocan (*p* = 0.010) and lower serum creatinine (*p* = 0.034) demonstrated significantly higher all-cause mortality. In our study, increased endocan and lower creatinine are associated with all-cause mortality in HD patients. Serum endocan levels could serve as a biomarker for a high mortality risk in HD patients.

## 1. Introduction

Patients with end-stage renal disease (ESRD) undergoing hemodialysis (HD) have higher rates of cardiovascular (CV) events and CV disease-related mortality [1,2]. Several risk factors, including hypertension, diabetes mellitus (DM), dyslipidemia, and calcium and phosphate disturbances, can contribute to CV mortality in ESRD patients [2,3]. In the pathophysiology of CV disease, endothelial dysfunction and inflammation play crucial roles as initial events in the progression of atherosclerosis and vascular calcification [4]. Several cytokines, including interleukin-1, interleukin-6, and tumor necrosis factor-α (TNF-α), are known to participate in endothelial inflammation. Some traditional biomarkers indicating endothelial inflammatory status include C-reactive protein, fibrinogen, serum amyloid A, E-selectin, P-selectin, vascular cell adhesion molecule-1, and intercellular adhesion molecule 1 [5]. Advanced molecular biology techniques have helped identify novel biomarkers that contribute to endothelial dysfunction, such as endocan, NGAL, PCSK9, and adiponectin. Endocan is a biomarker that demonstrates potential in representing endothelial activation.

Endocan, a soluble dermatan sulfate proteoglycan, is primarily secreted and expressed by activated endothelial cells in the lung and kidney [6,7]. It was up-regulated by pro-inflammatory cytokines, such as TNF-α and Interleukin-1β (IL-1β), and pro-angiogenic factors, such as vascular endothelial growth factor-A (VEGF-A) and fibroblast growth factor-2 (FGF-2) [6,8]. In cancer, endocan has been observed to participate in angiogenesis and tumor growth, indicating its potential as a tumor marker and a novel target for cancer therapy [7]. In transplanted kidneys, the pathologic scores of glomerulitis and peritubular capillarityis are associated with the severity of antibody-mediated rejection (ABMR) and were significantly higher in individuals with elevated serum and urine endocan levels. Endocan levels also have the potential to serve as markers for predicting worse renal survival [9]. Several studies have found that serum endocan positively correlates with various cardiovascular diseases, including hypertension, coronary artery disease, myocardial infarction, heart failure, and atherosclerosis [8,10,11,12,13,14,15]. It can also serve as a predictor for major adverse cardiovascular events after acute myocardial infarction or mortality in heart failure [12,14,15]. Elevated endocan levels have been observed in acute kidney injury, predicting the need for renal replacement therapy. Additionally, there is a positive correlation between the chronic kidney disease (CKD) stage and the severity of proteinuria. Studies have also reported a positive association between endocan levels and all-cause mortality as well as cardiovascular events in individuals with CKD [6,16]. In ESRD patients undergoing peritoneal dialysis, Peter et al. reported that a higher endocan level is associated with lower albumin, higher C-reactive protein levels, and lower CV event-free survival. However, there is no correlation between endocan levels and survival in patients treated by peritoneal dialysis [17]. Jin et al. reported endocan as a predictor of cardiovascular events in patients with ESRD on hemodialysis [18]. Due to the potential role of endocan in predicting outcomes in renal transplant, cardiovascular disease, and peritoneal dialysis patients in previous studies, our aim was to investigate the association between endocan levels and mortality in hemodialysis patients.

Additionally, the nutrient status of patients showed a strong correlation with their survival in hemodialysis. Nutrient markers, such as albumin, BMI, and creatinine, are also influenced by inflammation status, which was measured by CRP. Endocan, being a marker of vascular inflammation, was reported to have a negative correlation with albumin [17]. Therefore, we also collected commonly used and routinely tested markers, such as albumin, BMI, and creatinine, and investigated their relationship with endocan. Therefore, in this longitudinal observational study, we measured endocan levels, clinical characteristics, biochemistry data, and their association with patient mortality. Additionally, we examined the predictive value of endocan for all-cause mortality in hemodialysis patients.

## 2. Materials and Methods

### 2.1. Participants

This single-center observational longitudinal study was conducted from February to May 2014. It included participants aged 20 and above, who had been undergoing chronic hemodialysis for more than 3 months. The hemodialysis modality includes 4 h per session, three times a week, and the use of a high-flux polysulfone disposable dialyzer (FX class dialyzer; Fresenius Medical Care, Bad Homburg, Germany). A total of 103 HD patients in our HD room were enrolled in this study, all of whom provided informed consent before inclusion. This study was approved by the Research Ethics Committees of Hualien Tzu Chi Hospital, Buddhist Tzu Chi Medical Foundation (IRB103-136-B). Patients with acute infections, malignancies, amputations, acute heart failure, recent kidney transplants within the last 6 months, and a life expectancy of less than 6 months at the time of blood sampling were excluded from this study. Systolic and diastolic blood pressures were measured three times at 5 min intervals before the initiation of hemodialysis, and the values were averaged for further analysis. Patients with systolic blood pressure (SBP) ≥ 140 mmHg, and/or diastolic blood pressure (DBP) ≥ 90 mmHg, or those receiving any antihypertensive medications in the past 2 weeks, are defined as having hypertension. Patients with DM are identified by fasting plasma glucose levels of 126 mg/dL or higher, or by the use of oral hypoglycemic medications or insulin.

### 2.2. Anthropometric Analysis

Body height and weight were measured to the nearest half-centimeter and half-kilogram, respectively, with participants wearing light clothing and without shoes upon arrival at the HD room. Post-HD body weight was also measured. Pre-HD and post-HD body mass index (BMI) were calculated by dividing the pre-HD and post-HD body weights in kilograms by the square of height in meters, respectively.

### 2.3. Biochemical Investigations

Approximately 5 mL of blood samples were obtained before the start of hemodialysis in the midweek session. The hemoglobin level was determined using a Sysmex SP-1000i (Sysmex American, Mundelein, IL, USA). The remaining blood sample underwent centrifugation for biochemical analyses, encompassing total cholesterol (TCH), triglycerides (TG), glucose, blood urea nitrogen, creatinine, calcium, phosphorus, and albumin. These were analyzed using an autoanalyzer (Siemens Healthineers Headquarters, Siemens Healthcare GmbH, Henkestr, Erlangen, Germany) and a commercially available enzyme-linked immunosorbent assay. We utilized commercially available enzyme-linked immunosorbent assays to assess the serum levels of human endocan (Aviscera Bioscience, Inc., Santa Clara, CA, USA) and intact parathyroid hormone (iPTH; IBL International GmbH, Hamburg, Germany). Single-compartment dialysis urea kinetic model was used to calculate the fractional clearance index for urea (Kt/V) and the urea reduction ratio.

### 2.4. Follow-Up and Endpoints

All patients were tracked through medical records from the time they started HD until death or for 36 months until 30 June 2017. The endpoints of this study were defined as all-cause mortality. Event-free survival was defined as the duration between the enrollment evaluation and either the occurrence of the prespecified endpoint or the end of the study follow-up.

### 2.5. Statistical Analysis

The Kolmogorov–Smirnov test was used to analyze the normal distribution of continuous variables. Variables with a normal distribution were designated as means ± standard deviation and compared between groups using a two-tailed independent Student’s *t*-test. Variables not normally distributed were presented as medians and interquartile ranges (IQR), with comparisons between groups determined using the Mann–Whitney U-test (HD vintage, TG, glucose, iPTH, and endocan). Categorical variables were assessed using the Chi-square test and presented as numbers (percentages). Mortality predictive variables were identified through multivariate logistic regression analysis. The Kaplan–Meier curve depicted the survival curve for all-cause mortality in follow-up analyses. Patients were categorized into two groups based on the median serum endocan value (25.87 ng/mL). The cumulative proportion of patients free from all-cause mortality was compared using a log-rank test. Risk variables for all-cause mortality were explored using multivariate Cox regression models. Non-normally distributed continuous variables underwent natural logarithm (log) scale transformation for streamlined linear regression analysis. The correlation between log-endocan and biochemical parameters was analyzed using Pearson’s correlation analysis. The receiver operating curve (ROC) was used to calculate the area under the curve (AUC) to determine the optimal cutoff value of endocan for predicting all-cause mortality in HD patients. Data were analyzed using SPSS for Windows (version 19.0; SPSS Inc., Chicago, IL, USA). A *p* value < 0.05 was considered statistically significant.

## 3. Results

During the follow-up period, mortality was observed in 26 HD patients (25.2%), including hemorrhagic stroke (*n* = 6), ischemic stroke (*n* = 10), acute myocardial infarction (*n* = 5), and septic shock (*n* = 5). Table 1 summarizes the baseline clinical characteristics of the study population, categorized by mortality. The patients who died were older (*p* = 0.022), had a higher rate of DM (*p* = 0.018) and higher levels of serum endocan (*p* < 0.001), but had lower levels of serum albumin (*p* = 0.002) and serum creatinine (*p* < 0.001). There are no significant differences in HD vintage, hypertension rate, blood pressure level, BMI before and after HD, glucose, lipid profile, calcium and phosphate level, urea reduction rate, Kt/V, and antihypertensive or lipid-lowering drugs between HD patients with or without mortality after a 3-year follow-up period.

A Cox regression analysis was conducted to identify risk factors associated with mortality in HD patients (Table 2). In the analysis, age (hazard ratio (HR): 1.033, 95% confidence interval (CI), 1.002–1.066, *p* = 0.036), DM (HR: 2.645, 95% CI, 1.200–5.831, *p* = 0.016), albumin (HR: 0.247, 95% CI, 0.103–0.594, *p* = 0.002), creatinine (HR: 0.633, 95% CI, 0.509–0.788, *p* < 0.001) and endocan level (HR: 3.865 (95% CI, 1.548–9.649, *p* = 0.004) are associated with an increased risk of mortality in HD patients. After adjustment of the significant variables by multivariate Cox regression analysis, only the higher serum endocan level (adjusted HR (aHR): 3.420, 95% CI, 1.348–8.678, *p* = 0.010) and the lower serum creatinine level (aHR: 0.736, 95% CI, 0.554–0.977, *p* = 0.034) show a significant association with mortality. The use of angiotensin-receptor blocker, statin, and calcium-channel blocker showed no significant association with mortality (Supplement Table S1).

Table 3 displays the correlation between serum log-endocan and both clinical and biochemical variables. The results showed a significant negative association between serum log-endocan level and pre-HD BMI (r = −0.387, *p* < 0.001), post-HD BMI (r = −0.395, *p* < 0.001), log-TG (r = −0.207, *p* = 0.036), and creatinine (r = −0.337, *p* = 0.001).

**Figure 1:**
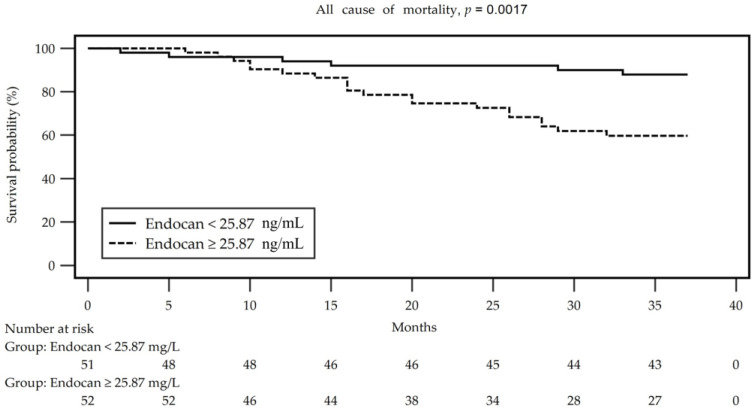
Kaplan–Meier analysis of endocan (divided into two groups according to value more and less than median value 25.87 ng/mL) for all-cause mortality of hemodialysis patients.

Figure 1 illustrates the survival probability based on serum endocan levels, dividing patients into two groups according to a value greater or less than the median value (25.87 ng/mL). Using a Kaplan–Meier curve, it was observed that a higher serum endocan level corresponded to a lower survival probability (*p* = 0.017), as determined by the log-rank test.

**Figure 2:**
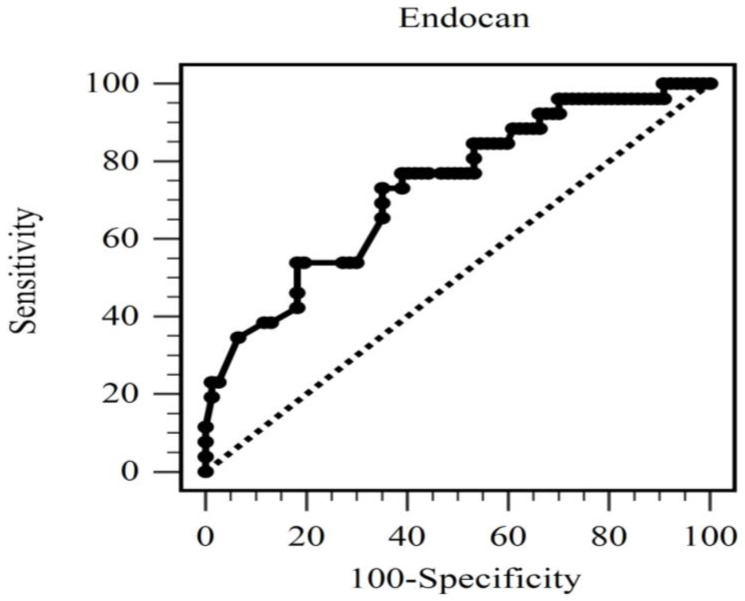
The area under the receiver operating characteristic curve indicates the diagnostic power of endocan level to predict all-cause mortality of hemodialysis patients.

ROC curve analysis suggested that the AUC for predicting mortality in HD patients, using an optimal serum endocan level cutoff point of 26.84 ng/mL, was 0.732 (95% CI, 0.636–0.815, *p* = 0.0001). The sensitivity and specificity were 73.08% and 64.94%, respectively (Figure 2).

## 4. Discussion

In the present study, we observed that patients undergoing hemodialysis with a higher risk of mortality exhibited a greater prevalence of diabetes mellitus, older age, elevated serum endocan levels, and lower serum creatinine and albumin levels. After adjusting for confounding factors through multivariate Cox regression analysis, a higher serum endocan level and a lower serum creatinine level persist in showing an association with mortality. However, there is no association observed with age, DM, albumin level, or the use of antihypertensive or lipid-lowering drugs.

Creatinine serves not only as a marker of renal function but also as an indicator of muscle mass and strength [19,20]. The association between creatinine, nutrient status, and its correlation with mortality has also been studied in previous research. The utilization of the modified creatinine index, in conjunction with the Geriatric Nutritional Risk Index (GNRI) for nutrient status assessment, has shown a strong correlation with mortality in hemodialysis patients [21,22,23,24,25,26]. As an indicator of poor nutrient status and reduced muscle mass, our study also demonstrates a greater risk of all-cause mortality in hemodialysis patients with lower creatinine levels before hemodialysis.

Albumin is another commonly used indicator of nutrient status in hemodialysis patients. Whether used alone or as part of GNRI, it shows a strong negative correlation with mortality in both hemodialysis and peritoneal dialysis patients [21,27,28,29]. Previous studies have also shown a negative correlation between endocan and GNRI [18]. However, the albumin level showed no correlation with mortality after adjusting with multivariate Cox regression analysis in our study. This may be due to an insufficient sample size or, although statistically significant, a small absolute difference between the mortality (4.25 ± 0.44 mg/dL) and no mortality (3.95 ± 0.41 mg/dL) groups.

Inflammation, as indicated by the use of CRP as a marker, had a negative effect on muscle mass and BMI in hemodialysis patients [30,31]. Endocan, although not commonly used, is a marker representing vascular inflammation. In peritoneal dialysis, a high endocan level has been reported to be negatively correlated with nutrient status, including albumin level, subjective global assessment, and malnutrition inflammation score [32]. In our study, we demonstrated a negative correlation between serum log-endocan levels and BMI, as well as creatinine, suggesting a potential relationship between endocan and muscle mass in hemodialysis patients. This may serve as a marker for investigating the relationship between vascular inflammation and nutrient status or muscle mass, but further study is needed.

Endocan, a novel proteoglycan primarily secreted by vascular endothelium, was first reported by Lassale et al. in 1996 [33]. It was highly restrictedly distributed to vascular endothelial cells and initially named human endothelial cell-specific molecule-1 [33]. However, further research confirmed that it belongs to the proteoglycan family and is termed endocan. Endocan is also expressed in tissues or cells with active proliferation, such as glandular tissue, bronchial epithelium, germinal centers of lymph nodes, cardiomyocytes, liver cells, and neurons [8]. Unlike other proteoglycans that contain large molecules and several glycosaminoglycan chains, endocan has a molecular mass of only 20 kDa and contains only a single dermatan sulfate chain. Unlike other proteoglycans, Endocan does not provide structural support. Instead, it is a secretory molecule that modulates many biological processes, including cell adhesion, proliferation, and neovascularization [34,35]. During acute infection, macrophages secrete TNF and IL-1. These stimulate the endothelium to express cell adhesion molecules (CAMs) such as E- and P-selectin, and intercellular adhesion molecule-1 (ICAM-1). These CAMs can interact with the ligand, lymphocyte function-associated antigen-1 (LFA-1), on the leukocytes, initiating leukocyte transmigration and migration to the inflammatory site. The binding of endocan with LFA-1 induced a negative effect on LFA-1 interaction with ICAM-1. Thus, endocan functions as an inhibitor of leukocyte homing and transmigration [8,34,35]. On the other hand, endocan is up-regulated by several pro-inflammatory cytokines, such as TNF-α, IL-1, and VEGF. In some reports, endocan has been found to up-regulate CAMs that express or activate the nuclear factor-kappa B pathway, leading to further inflammatory reactions. This distinct role may be attributed to the fact that the binding site of endocan can be regulated by divalent ions, such as Ca^2+^, Mg^2+^, or Mn^2+^ [8]. Endocan has also been found to preferentially express on a group of cells known as “tip cells”, located at the end of a developing vessel, functioning as sensors and mediating vascular growth. Its association with the neovascularization of tumors, the angiogenic switch in stem cells, and the endothelial-mesenchymal transition process, such as arterial wall remodeling, has been proven [34]. Endocan has also been studied in several kidney diseases such as acute kidney injury (AKI), CKD, and renal replacement therapy (RRT). In patients with acute respiratory distress syndrome who require RRT, serum endocan levels were found to be higher than in those who did not require RRT. The combined assessment of creatinine and endocan demonstrates better prognostic value in predicting the need for RRT than relying on creatinine alone [6]. In patients diagnosed with AKI, elevated endocan levels were also observed, and a higher plasma endocan cleavage ratio was correlated with an increased renal SOFA score [6,35]. In CKD, elevated levels of endocan were observed compared to the control group, and the concentration of endocan positively correlated with the CKD stage while negatively correlated with the estimated glomerular filtration rate [6]. Additionally, higher levels of endocan were noted in CKD patients with cardiovascular disease (CVD) than in those without CVD [36]. In hemodialysis, patients with higher endocan levels were found to have more frequent CV events, lower BMI, and lower serum TG and albumin levels [18]. In Chinese patients undergoing peritoneal dialysis, endocan levels were negatively associated with nutrient status. Higher endocan levels were associated with lower nutrition assessments by subjective goal assessment, higher malnutrition-inflammation scores, and lower serum albumin levels [17]. In peritoneal dialysis, the serum endocan level was also identified as a predictor of decreased urine volume, with higher levels noted in the group experiencing a more rapid decrease (>200 mL/year) [32]. In our study, we demonstrated that hemodialysis patients with mortality had significantly higher serum endocan levels. Using ROC curve analysis, we identified a cutoff value of 26.84 ng/mL for predicting mortality in hemodialysis patients. A negative correlation of serum log-endocan with BMI and serum creatinine was also noted. Unlike albumin or creatinine, which are used from a nutritional perspective to predict mortality in hemodialysis, endocan provides a pathogenic view to elucidate higher cardiovascular-related mortality in hemodialysis patients. This perspective may assist in identifying a high-risk mortality group of patients who may benefit from more intense CVD control, such as strict management of serum calcium and phosphate, dry body weight, blood pressure, or cholesterol levels.

Our present study has several limitations. Firstly, we excluded conditions that may induce an inflammatory state, such as infection, malignancy, and recent surgery. We checked the endocan level before hemodialysis to prevent dialysis-related endocan fluctuation [37]. However, the endocan level was measured only once after the patient was included, so the trend of endocan level change was not known. This could potentially lead to incorrect patient classification. Secondly, several medications, such as statins, amlodipine, valsartan, and vitamin D, have been reported to decrease endocan levels [35]. These medications are commonly used in hemodialysis patients, but not every patient uses the same medication. Additionally, the variation in the time from when a patient takes the drug to when the patient comes to the HD room for blood sampling may all affect endocan levels. Thirdly, the cutoff value of endocan was calculated using only 103 patients. A larger sample size is needed in further studies to determine the reliability of the cutoff value.

## 5. Conclusions

Endocan is a novel marker representing endothelial dysfunction and inflammation. Its correlation with cardiovascular disease and its potential as an outcome predictor has been demonstrated in the general population, CKD, and ESRD patients. In our study, we established a correlation between higher endocan levels and all-cause mortality in hemodialysis patients. We also identified an optimal cutoff value of 26.84 ng/mL, with a sensitivity of 73.08% and specificity of 64.94%. Additionally, we observed a negative correlation between serum log-endocan levels and BMI, as well as creatinine. This finding may help us to classify the risk of hemodialysis patients.

## Figures and Tables

**Table 1:** Clinical variables of the hemodialysis patients with or without mortality.

Variables	All Participants (*n* = 103)	Participants without Mortality (*n* = 77)	Participants with Mortality (*n* = 26)	*p* Value
Age (years)	62.60 ± 13.14	60.88 ± 13.30	67.69 ± 11.44	0.022 *
Female, *n* (%)	52 (50.5)	39 (50.6)	13 (50.0)	0.954
Diabetes mellitus, *n* (%)	43 (41.7)	27 (35.1)	16 (61.4)	0.018 *
Hypertension, *n* (%)	59 (57.3)	42 (45.5)	17 (65.4)	0.334
HD vintage (months)	55.32 (22.68–117.84)	56.88 (21.42–131.40)	46.38 (25.35–81.21)	0.611
Pre-HD BMI (kg/m^2^)	24.85 ± 4.63	25.26 ± 4.56	23.66 ± 4.72	0.129
Post-HD BMI (kg/m^2^)	24.01 ± 4.44	24.39 ± 4.44	22.90 ± 4.61	0.148
Inter-dialytic weight gain (kg)	2.66 ± 1.54	2.61 ± 1.63	2.8 ± 1.27	0.576
Systolic blood pressure (mmHg)	144.24 ± 26.75	143.12 ± 26.70	147.58 ± 27.13	0.465
Diastolic blood pressure (mmHg)	77.72 ± 15.91	78.27 ± 16.26	76.08 ± 15.01	0.545
Endocan (ng/mL)	25.87 (14.64–39.69)	21.93 (11.88–32.17)	38.87 (24.47–56.32)	<0.001 *
Hemoglobin (g/dL)	10.48 ± 1.19	10.44 ± 1.23	10.63 ± 1.07	0.469
Total cholesterol (mg/dL)	147.45 ± 35.64	150.36 ± 36.12	138.81 ± 33.38	0.154
Triglyceride (mg/dL)	115.00 (87.00–178.00)	118.00 (92.50–193.00)	104.00 (81.25–133.25)	0.102
Albumin (g/dL)	4.18 ± 0.45	4.25 ± 0.44	3.95 ± 0.41	0.002 *
Glucose (mg/dL)	131.00 (106.00–163.00)	129.00 (103.00–159.00)	139.00 (117.50–176.50)	0.158
Blood urea nitrogen (mg/dL)	60.89 ± 13.40	61.57 ± 13.03	58.88 ± 14.53	0.379
Creatinine (mg/dL)	9.39 ± 1.98	9.85 ± 1.84	8.05 ± 1.79	<0.001 *
Total calcium (mg/dL)	9.01 ± 0.79	9.05 ± 0.78	8.90 ± 0.82	0.431
Phosphorus (mg/dL)	4.65 ± 1.34	4.73 ± 1.35	4.41 ± 1.32	0.295
iPTH (pg/mL)	197.00 (69.30–453.80)	211.70 (71.80–457.55)	173.10 (57.30–416.23)	0.873
Urea reduction rate	0.74 ± 0.04	0.73 ± 0.04	0.74 ± 0.05	0.352
Kt/V (Gotch)	1.35 ± 0.17	1.34 ± 0.16	1.37 ± 0.18	0.347
ARB, *n* (%)	33 (32.0)	25 (32.5)	8 (30.8)	0.873
β-blocker, *n* (%)	35 (34.0)	28 (36.4)	7 (26.9)	0.380
CCB, *n* (%)	43 (41.7)	34 (44.2)	9 (34.6)	0.394
Statin, *n* (%)	16 (15.6)	14 (18.2)	2 (7.7)	0.202
Fibrate, *n* (%)	13 (12.6)	9 (11.7)	4 (15.4)	0.624

Values for continuous variables given as means ± standard deviation and compared by Student’s *t*-test; variables not normally distributed (given as medians and interquartile range and compared by Mann–Whitney U test; values are presented as number (%), and analysis was performed using the chi-square test. HD: hemodialysis; BMI: body mass index; iPTH: intact parathyroid hormone; Kt/V: fractional clearance index for urea; ARB: angiotensin-receptor blocker; CCB: calcium-channel blocker. * *p* < 0.05 was considered statistically significant.

**Table 2:** Risk factor of mortality of the 103 hemodialysis patients.

Variables	HR	95% CI	*p* Value	aHR	95% CI	*p* Value
Age (years)	1.033	1.002–1.066	0.036 *	1.026	0.995–1.058	0.104
Female/Male	1.002	0.465–2.163	0.995	–	–	–
Diabetes mellitus	2.645	1.200–5.831	0.016 *	2.003	0.847–4.738	0.114
Hypertension	1.582	0.705–3.549	0.266	–	–	–
HD vintage (months)	0.996	0.990–1.003	0.273	–	–	–
Pre-HD BMI (kg/m^2^)	0.953	0.874–1.039	0.274	–	–	–
Post-HD BMI (kg/m^2^)	0.942	0.859–1.032	0.198	–	–	–
Inter-dialytic weight gain (kg)	1.117	0.823–1.447	0.401			
Systolic blood pressure (mmHg)	1.006	0.992–1.020	0.412	–	–	–
Diastolic blood pressure (mmHg)	0.994	0.970–1.019	0.636	–	–	–
Endocan						
<25.87 ng/mL	1			1		
≥25.87 ng/mL	3.865	1.548–9.649	0.004 *	3.420	1.348–8.678	0.010 *
Hemoglobin (g/dL)	1.147	0.831–1.583	0.404	–	–	–
Total cholesterol (mg/dL)	0.991	0.979–1.003	0.136	–	–	–
Triglyceride (mg/dL)	0.996	0.990–1.001	0.115	–	–	–
Albumin (g/dL)	0.247	0.103–0.594	0.002 *	0.751	0.252–2.238	0.607
Glucose (mg/dL)	1.004	0.999–1.009	0.157	–	–	–
Blood urea nitrogen (mg/dL)	0.987	0.960–1.015	0.374	–	–	–
Creatinine (mg/dL)	0.633	0.509–0.788	<0.001 *	0.736	0.554–0.977	0.034 *
Total calcium (mg/dL)	0.818	0.500–1.339	0.424	–	–	–
Phosphorus (mg/dL)	0.854	0.637–1.143	0.288	–	–	–
iPTH (pg/mL)	1.001	0.999–1.002	0.356	–	–	–
Urea reduction rate (×100)	1.041	0.951–1.139	0.389	–	–	–
Kt/V (Gotch)	2.579	0.291–22.838	0.395	–	–	–
ARB	0.937	0.408–2.155	0.879	–	–	–
β-blocker	0.721	0.303–1.717	0.460	–	–	–
CCB	0.453	0.182–1.127	0.089	–	–	–
Statin	0.460	0.109–1.946	0.291	–	–	–
Fibrate	1.237	0.426–3.590	0.696	–	–	–

HR: hazard ratio; CI: confidence interval; aHR: adjusted hazard ratio; HD: hemodialysis; BMI: body mass index; iPTH: intact parathyroid hormone; Kt/V: fractional clearance index for urea; ARB: angiotensin-receptor blocker; CCB: calcium-channel blocker. * *p* < 0.05 was considered statistically significant after Cox regression analysis. Multivariate Cox proportional-hazards regression analysis is adjusted for age, diabetes mellitus, albumin, creatinine, and endocan.

**Table 3:** Spearman correlation coefficients between serum log-transformed endocan levels and clinical variables.

Variables	Spearman Coefficient of Correlation	*p* Value
Age (years)	0.060	0.545
Log-HD vintage (months)	0.060	0.549
Pre-HD BMI (kg/m^2^)	–0.387	<0.001 *
Post-HD BMI (kg/m^2^)	–0.395	<0.001 *
Systolic blood pressure (mmHg)	0.030	0.760
Diastolic blood pressure (mmHg)	–0.095	0.339
Hemoglobin (g/dL)	–0.085	0.392
Total cholesterol (mg/dL)	0.019	0.853
Log-Triglyceride (mg/dL)	–0.207	0.036 *
Albumin (g/dL)	–0.162	0.102
Log-Glucose (mg/dL)	–0.114	0.251
Blood urea nitrogen (mg/dL)	0.012	0.908
Creatinine (mg/dL)	–0.337	0.001 *
Total calcium (mg/dL)	–0.075	0.449
Phosphorus (mg/dL)	–0.118	0.234
Log-iPTH (pg/mL)	–0.096	0.337
Urea reduction rate	0.099	0.318
Kt/V (Gotch)	–0.098	0.323

Data of endocan, HD vintage, triglyceride, glucose, and iPTH levels showed skewed distribution and therefore were log-transformed before analysis. Analysis of data was performed using the Spearman correlation analysis. HD: hemodialysis; BMI: body mass index; iPTH: intact parathyroid hormone; Kt/V: fractional clearance index for urea. * *p* < 0.05 was considered statistically significant.

## Data Availability

The data presented in this study are available on request from the corresponding author.

## Supplementary Materials

The following supporting information can be downloaded at: https://www.mdpi.com/article/10.3390/jcm12237427/s1, Table S1: Multivariate regression analysis of related risk of mortality.
