# Supplementary material for: Endocan as a Potential Marker for Predicting All-Cause Mortality in Hemodialysis Patients
Source: J Clin Med. 2023 Nov 30;12(23):7427. doi: 10.3390/jcm12237427 (PMC10706971; doi:10.3390/jcm12237427)
Supplement: Supplementary file 1 [file jcm-12-07427-s001.zip › jcm-2693516-supplementary.pdf]

Supplement Table S1. multivariate regression analysis of related risk of mortality

variables	**odds ratio**	**95% CI**	***p value***
albumin	0.813	0.251-2.631	0.730
creatinine	0.751	0.557-1.014	0.062
Sex	1.487	0.616-3.589	0.377
DM	1.760	0.738-4.199	0.202
Endocan	3.870	1.458-10.268	0.007*
age	1.037	1.000-1.076	0.051
ARB	1.539	0.558-4.100	0.389
Statin	0.504	0.116-2.192	0.361
CCB	0.481	0.173-1.338	0.161

DM: diabetic mellitus; ARB: angiotensin receptor blocker; CCB: calcium channel blocker. * $p < 0.05$ was considered statistically significant.
